# Implementation of Evidence-Based International Recommendations Reduces Postoperative Delirium Rate in Patients Undergoing Cardiac Surgery or Interventions: A System-Based Quality Improvement Study

**DOI:** 10.31083/j.rcm2510369

**Published:** 2024-10-16

**Authors:** Simon Milz, Caroline Holaubek, Jan Siebel, Nikolai Hulde, Franziska Wefer, Andreas Fruend, Katharina Tigges-Limmer, Jan Gummert, Vera von Dossow

**Affiliations:** ^1^Institute of Anaesthesiology and Pain Therapy, Heart and Diabetescenter Bad Oeynhausen, NRW, Ruhr-University Bochum, 32545 Bad Oeynhausen, Germany; ^2^Department of Anaesthesia, Intensive Care Medicine and Pain Medicine, Medical University of Vienna, 1090 Wien, Austria; ^3^Nursing Development, Management of Nursing, Heart and Diabetescenter Bad Oeynhausen, NRW, Ruhr-University Bochum, 32545 Bad Oeynhausen, Germany; ^4^Department of Physiotherapy, Heart and Diabetescenter Bad Oeynhausen, NRW, Ruhr-University Bochum, 32545 Bad Oeynhausen, Germany; ^5^Clinic for Thoracic and Cardiovascular Surgery, Heart and Diabetescenter Bad Oeynhausen, NRW, Ruhr-University Bochum, 32545 Bad Oeynhausen, Germany

**Keywords:** delirium, cardiac surgery, perioperative care, postoperative care

## Abstract

**Background:**

Delirium is a frequent and serious complication of cardiac procedures that can lead to serious long-term health restrictions. As primary prevention is more effective in reducing rate of delirium than the therapy itself, this study aimed to investigate the effect of a multidisciplinary delirium prevention bundle on the postoperative delirium rate in patients undergoing cardiac procedures.

**Methods:**

In this system-based quality improvement study, a four-component delirium prevention bundle was implemented in patients undergoing cardiac procedures at a single high-volume center. The program included preoperative delirium risk stratification, multidisciplinary education of consensus guidelines, written memory aids, and post-anesthetic visits with delirium screening until postoperative day three.

**Results:**

Overall, 234 patients were included and analyzed during the 6-month study period. The overall delirium incidence rate was 12.4%. After the first 3-month baseline implementation period, the delirium rate was 17.2%, compared with 7.6% (*p* = 0.026) after implementation of the delirium prevention bundle. Multivariate analysis revealed independent risk factors such as age [adjusted odds ratio (OR): 1.046; 95% confidence interval (CI): 1.002–1.092; *p* = 0.042], double valve surgery [adjusted OR: 13.1; 95% CI: 3.240–52.974; *p* < 0.0001], and peripheral artery disease [adjusted OR: 8.131; 95% CI: 2.336–28.306; *p* < 0.001]. Hospital stay was significantly longer in patients with delirium [median 13 (12–19.5) versus 12 (11–14) days, *p* = 0.009].

**Conclusions:**

This multidisciplinary system-based quality improvement study demonstrated a beneficial effect on the delirium rate after the implementation of a four-component delirium prevention bundle in patients undergoing cardiac surgery or intervention. Furthermore, multivariate analysis revealed important independent risk factors for delirium development. This might improve early risk stratification and strategies for this high-risk patient collective.

## 1. Introduction 

Delirium is a form of acute brain failure characterized by altered consciousness with a reduced ability to focus, sustain, or shift attention. It develops quickly and tends to fluctuate over the day [1]. Currently, the pathophysiology of delirium is not well understood, which likely contributes to the current state of low-quality evidence for perioperative guidelines. Postoperative delirium is a common and harrowing complication in older surgical patients. It is hypothesized that delirium can accelerate cognitive decline and the onset of dementia, or even worsen the severity of dementia [1].

Delirium is a common phenomenon observed on the first postoperative day. Delirium rate in patients in the intensive care unit (ICU) was 45–50% in former studies [1, 2, 3]. Ventilated patients on ventilation in the ICU had a higher incidence of delirium. 80% of these patients experience at least a short delirious period [2, 4]. Being delirious can be the most distressing aspect of the perioperative experience for patients and their families. Furthermore, postoperative delirium is associated with increased mortality, cognitive and functional decline, increased hospital length of stay, and substantial annual health-care costs [1, 2].

Although delirium awareness has grown in recent years owing to announcements by different medical societies as well as publications of evidence-based international guidelines [5, 6], regular screening and monitoring with a validated delirium screening tool has not yet been implemented in the majority of hospitals [7].

Recent prevention trials involving pharmacological strategies such as ketamine and dexmedetomidine have demonstrated inconsistent findings. In contrast, non-pharmacological multicomponent initiatives such as the Hospital Elder Life Program have consistently reduced the incidence and burden of delirium across various hospital settings [1]. However, a substantial proportion of delirium cases are not prevented; therefore, effective prevention and management strategies are needed to complement multicomponent nonpharmacological therapies.

Delirium screening and monitoring may reduce the severity and duration of delirium as well as in-hospital mortality [8, 9]. Therefore, early risk stratification and delirium prevention strategies are crucial for improved outcomes [10]. Many non-pharmacological prevention measures have shown significant effects in patients undergoing cardiac surgery [10, 11]. In the case of a multifactorial etiology of delirium, the aim is to use multicomponent programs to detect several known risk factors for delirium and counteract them as preventively as possible [1].

In contrast to postoperative delirium, early postoperative cognitive decline occurs at the end of the first week after cardiac surgery and does not affect consciousness. Nevertheless, it is associated with markedly adverse outcomes and the duration of postoperative cognitive decline is significantly prolonged. Since cerebral inflammation is an important factor in the pathogenesis of delirium, preoperative administration of corticosteroids may reduce the incidence of postoperative cognitive decline after surgery on cardiopulmonary bypass [12, 13].

Despite strong evidence for delirium prevention tools in cardiac surgery, their implementation in routine clinical practice remains rare. Therefore, the primary aim of this prospective system-based quality improvement study was to determine the incidence of delirium before and after the implementation of a four-component delirium prevention bundle. The secondary aim was to identify significant independent risk factors in high-risk patients undergoing cardiac surgery or interventions at a single high-volume center.

## 2. Methods

### 2.1 Patients

For our system-based quality improvement study, we included patients aged >18 years who underwent cardiac surgery or interventional cardiology procedures at the Heart and Diabetes Center North Rhine Westfalia (NRW) between June 2019 and December 2019. Patients who underwent coronary artery bypass grafting, valve surgery, aortic surgery, left ventricular assist device (LVAD) implantation, or transcatheter aortic valve implantation (TAVI) were included. Patients who were incapable of providing consent or were unable to perform the tests due to a lack of German language skills were excluded from the study. Patients and visitors received a flyer containing information about the multidisciplinary delirium bundle, including contact options for further questions or feedback.

### 2.2 Ethical Statement

The study was approved by the local ethics committee of Ruhr-University Bochum at HDZ NRW Ostwestfalen on 5th June 2019 (ID: DEL-I: 2019-506).

All patients were verbally informed by the investigator the day before the operation or interventional procedure as part of the premedication visit and were required to confirm their participation in the study in writing after receiving patient information for documentation and evaluation of their data. All patients were preoperatively Confusion Assessment Method- Intensive Care Unit (CAM-ICU)-negative.

### 2.3 Intervention Design and Implementation Strategy

This prospective system-based quality improvement study was intended to accompany the implementation of delirium management at the Heart and Diabetes Center NRW in cardiac surgery and cardiology patients from June 2019 to December 2019. The project plan was divided into four phases.

Phase I (Jan 2019–April 2019) of the project included several kick-off meetings to establish and discuss the need for the project, overarching delirium management, and forming an interdisciplinary core team. The task of the core team was to define the screening instruments to be used and to develop preventive non-pharmacological and pharmacological strategies, as well as evidence-based treatment regimens. By consensus, the core team determined in Phase I that valid screening instruments, such as the CAM-ICU and a monitoring system, should be used consistently. The core team consisted of anesthesiologists, intensive care medicine, nursing staff, medical psychology, neurology, cardiology, cardiac surgery, physiotherapy, pharmacy, and information technology (IT) specialists.

In Phase II (April 2019–August 2019), several training measures were introduced in our center: multiprofessional education for selected medical and nursing staff members to ensure knowledge transfer to their teams, a symposium on the topic of delirium as an interdisciplinary training event, and flyers for general wards and intensive care units to raise awareness of the topic. Simultaneously, a multicomponent program for the prevention of postoperative delirium was established.

Components of our non-pharmacological delirium management:

Our delirium management comprised four non-pharmacological components:

(1) Introduction of preoperative screening for delirium predisposing factors;

(2) Consistent post-anesthesia ward rounds with delirium screening until postoperative day 3;

(3) Multidisciplinary education on consensus guidelines [5, 6];

(4) Written memory aids in the form of flyers.

Measures for delirium prevention, such as early mobilization, avoiding premedication with benzodiazepines, daily pain assessment and adequate treatment, avoiding malnutrition, the use of hearing or visual aids for better orientation, and noise reduction, were part of a multidisciplinary education program. Furthermore, all the participants were educated on delirium screening and documentation under supervision.

In Phase III (June 2019–August 2019), the delirium rate was surveyed for three months before the implementation of delirium management. We set August as the month of implementation completion.

In Phase IV (September 2019–December 2019), all staff were brought to approximately the same level of information so that screening tools could be used and documented in electronic patient data management systems. In these three months, the delirium rate after implementation was collected for comparison.

### 2.4 Data Collection and End Points

During the premedication visit, patients were screened for delirium-predisposing factors [1]. These factors include neurodegenerative or mental illnesses, chronic pain, hearing and visual impairments, immobility, and substance abuse. An age >70 years is also considered a presumed risk factor for delirium. Patients with at least three risk factors were assumed to have an increased risk of delirium. Additionally, certain comorbidities were recorded, including arterial hypertension, diabetes mellitus, atrial fibrillation, cerebral stroke, and peripheral artery disease. The determination of the postoperative nausea and vomiting (PONV) score to estimate the individual risk of nausea and vomiting after surgical intervention was also part of the premedication visit.

Following surgery or intervention, a three-day post-anesthesia visit was conducted by the study team on postoperative day 1 to collect data for the study. For this purpose, delirium screening using the CAM-ICU, pain measurement using the Numeric Rating Scale (NRS), and measurement of the degree of sedation (RASS) were performed and documented in the intensive care unit and general ward. The CAM-ICU represents the endpoint of our study. For our study, we used the German version of the CAM-ICU for ventilated and non-ventilated patients in an intensive care unit, which, according to Guenther *et al*. [14], has a sensitivity of 88–92% and a specificity of 100%.

### 2.5 Surgical and Anesthetic Procedures

All patients received morphine 5 mg and droperidol 1.25 mg subcutaneously as premedication on the day of surgery. In the subsequent induction of anesthesia, in most cases, the usual extended monitoring for cardiac surgery was used, consisting of electrocardiogram (ECG), peripheral oxygen saturation, invasive blood pressure measurement, and a central and peripheral venous catheter. The patients received sufentanil 0.5 µg/kg body weight (BW), etomidate 0.15–0.3 mg/kg BW, rocuronium 0.5 mg/kg BW, dexmedetomidine bolus 0.2–0.6 µg/kg BW (partially given; increasingly used in the period after the implementation of delirium management) and hydrocortisone 100 mg intravenously as a general anesthetic via peripheral venous access. This procedure was followed by endotracheal intubation. Anesthesia was maintained by continuous infusion of remifentanil (0.5–1 µg/kg/min) and vaporization of sevoflurane 1–1.5 vol% and additively dexmedetomidine according to hospital standards. Postoperatively, all patients were sedated, intubated, ventilated, and transferred to the intensive care unit according to internal hospital standards. The patients who underwent TAVI were treated with propofol and dexmedetomidine. None of the patients received additional regional anesthesia.

### 2.6 Statistical Analysis

Differences in delirium rates were assessed using the chi-squared test. The group of patients with postoperative delirium during their hospital stay was compared with the group of patients without delirium. Bivariate and multivariate risk factors for in-hospital delirium after cardiac surgery or intervention were determined in all patients included in the study. Categorical variables were compared using the chi-square and Fisher’s exact tests. Continuous variables were compared using the Mann–Whitney U test. A final multivariate logistic regression model was developed using stepwise backward regression analysis. Risk factors were considered statistically significant at *p* < 0.05. Data in the tables are presented as medians with 25% and 75% quartiles, unless otherwise stated. Analyses were performed using IMB SPSS Statistics software (version 27.0; IBM Corp., Armonk, NY, USA).

## 3. Results

### 3.1 Patient Characteristics

Finally, 383 patients who underwent cardiac surgery or interventional heart procedures were screened. However, six patients were incapable of obtaining consent due to severe dementia or depression and five due to insufficient German language skills. 27 patients refused to participate. After obtaining verbal and written informed consent, 345 patients were included in the study. All the patients met the inclusion criteria. A total of 111 patients were excluded from the study, where 74 were excluded owing to cancelled/postponed surgery caused by medical or organizational reasons. Additionally, 32 patients were excluded because of continuous psychiatric non-evaluability due to ongoing deep sedation or reintubation within the first 3 days after surgery or intervention. Five patients were transferred to another hospital within this period. Ultimately, 234 patients were included in the data analysis.

### 3.2 Incidence of Delirium

Postoperative delirium in a patient on at least one of the three postoperative days was detected in 29 cases with a positive CAM-ICU. Thus, the incidence of delirium in our study was 12.4%. The mean duration of delirium was 1.7 days. Delirium occurred primarily on the first postoperative day (11.9%). Thereafter, the incidence of delirium decreased steadily from day 2 (5.6%) to day 3 (2.6%) postoperatively. After the first 3-month baseline implementation period delirium rate was 17.2%, compared with 7.6% (*p* = 0.026) after implementation of the delirium prevention bundle.

### 3.3 Predisposing Factors for Delirium

When looking at the demographics (Table 1), a significant correlation was found between delirium occurrence and age (*p* = 0.002). The patients with delirium had a higher median age (72 years) than those without delirium (66 years). There were no statistically significant differences in the other parameters of the screened predisposing factors for delirium (Table 1).

**Table 1.** **Demographic and delirium risk scores**.

Parameter		Study population (n)	Delirium (n)	No delirium (n)	*p*-value
Age (years)		66.50 (58/75)	72 (66.5/78.5)	66 (57.0/74.0)	0.002
Sex		f = 62 (26.5%)	f = 7 (24.1%)	f = 55 (26.8%)	0.833
		m = 172 (73.5%)	m = 22 (75.9%)	m = 150 (73.2%)	
BMI (kg/m²)		27.4 (24.4/30.5)	27.4 (24.4/31.4)	27.5 (34.3/30.5)	0.837
Body height (cm)		173.0 (167.0/180.0)	172.0 (164.5/177.5)	174.0 (167.0/180.0)	0.399
Bodyweight (kg)		82.0 (73.0/94.0)	85 (70/94.5)	82.0 (73.0/93.5)	0.896
EuroSCORE II		1.8 (1.0/3.6)	3.9 (1.8/6.1)	1.65 (0.9/3.2)	0.151
NYHA		2 (2/3)	3 (2/3)	2 (2/3)	0.094
COPD		20 (8.5%)	4 (13.8%)	16 (7.8%)	0.297
Myocardial infarction		34 (14.5%)	1 (3.4%)	33 (16.1%)	0.066
Hospital length of stay (days)		12.0 (11.0/14.0)	13 (12/19.5)	12 (11/14)	0.009
ICU length of stay (days)		0.9 (0.8/1.1)	1.2 (0.8/1.2)	0.9 (0.8/1.0)	0.080
In-hospital mortality		1 (0.4%)	0 (0.0%)	1 (0.5%)	0.706
30-day mortality		1 (0.4%)	0 (0.0%)	1 (0.5%)	0.706
6-month mortality		6 (2.6%)	2 (6.9%)	4 (2.0%)	0.115
1-year mortality		11 (4.7%)	4 (13.8%)	7 (3.4%)	0.013
Delirium predisposing factors					
	Age >70 years old	100 (42.7%)	19 (65.5%)	81 (39.5%)	0.008
	Arterial hypertension	192 (82.1%)	25 (86.2%)	167 (81.5%)	0.599
	Diabetes mellitus	56 (23.9%)	9 (31.0%)	47 (22.9%)	0.361
	Atrial fibrillation	64 (27.4%)	10 (34.5%)	54 (26.3%)	0.383
	Cerebral stroke	5 (2.1%)	0 (0.0%)	5 (2.4%)	0.393
	Peripheral artery disease	18 (7.7%)	6 (20.7%)	12 (5.9%)	0.060
	Neurodegenerative/mental disease	28 (12.0%)	2 (6.9%)	26 (12.7%)	0.361
	Chronic pain patient	3 (1.3%)	0 (0.0%)	3 (1.5%)	0.550
	Hearing/visual impairment	42 (18.0%)	5 (17.2%)	37 (18.0%)	0.734
	Immobility	15 (6.4%)	2 (6.9%)	13 (6.3%)	0.702
	Substance abuse	4 (1.7%)	0 (0.0%)	4 (2.0%)	0.489

f, female; m, male; BMI, body mass index; ICU, intensive care unit; NYHA, New York Heart Association; COPD, chronic obstructive lung disease.

Preoperatively, a total of 24 (10.3%) patients had at least three predisposing factors for delirium; and therefore were classified as being at risk of delirium. An age >70 years was considered a prerequisite for this. Overall, however, only 6 (25.0%) of the 24 patients showed a positive CAM-ICU. According to the chi-square test, there was no significant correlation between the screened delirium-predisposing factors and positive CAM-ICU results (*p* = 0.122).

### 3.4 Surgery and Interventions

Regarding the types of surgery and interventions, some significant differences were observed in the comparison of delirious and non-delirious patients, although the surgical procedures were similar before and after the implementation of the delirium bundle. The operation of a bypass in combination with the mitral valve (coronary artery bypass grafting (CABG) + mitral valve surgery) (*p* = 0.028), as well as the operation of different valves simultaneously (*p* = 0.03), showed a higher incidence of delirium. In contrast, patients who underwent minimally invasive mitral valve surgery had a significantly lower incidence of postoperative delirium (*p* = 0.047). There were no significant differences in intraoperative time between the delirium and non-delirium groups. The standard approach for these cardiac operations was median sternotomy, which was performed in approximately two-thirds of all cases. Minimally invasive procedures, vascular access, and partial sternotomy were performed in descending order. There were no significant differences in the incidence of delirium between groups. Cardiopulmonary bypass during coronary artery bypass grafting did not result in a significant difference in the occurrence of delirium (Table 2).

**Table 2.** **Surgical procedures and specifics**.

Parameter	Study population (n)	Delirium (n)	No delirium (n)	*p*-value
CABG total	74 (31.6%)	7 (24.1%)	67 (32.7%)	0.354
CABG – cardiopulmonary bypass	13 (5.6%)	2 (6.9%)	11 (5.4%)	0.736
Off-pump CABG	61 (26.1%)	5 (17.2%)	56 (27.3%)	0.247
CABG + Valve	25 (10.7%)	3 (10.3%)	22 (10.7%)	0.571
CABG + Double valve surgery	1 (0.4%)	0 (0.0%)	1 (0.5%)	0.706
CABG + Mitral valve surgery	8 (3.4%)	3 (10.3%)	5 (2.4%)	0.028
CABG + Aortic valve surgery	16 (6.8%)	0 (0.0%)	16 (7.8%)	0.119
Valve total	110 (47.0%)	13 (44.8%)	97 (47.3%)	0.801
Valve – cardiopulmonary bypass	85 (36.3%)	9 (31.0%)	76 (37.1%)	0.527
Double valve surgery	13 (5.6%)	5 (17.2%)	8 (3.9%)	0.030
Mitral valve minimally invasive surgery	25 (10.7%)	0 (0.0%)	25 (12.2%)	0.047
Mitral valve surgery	12 (5.1%)	2 (6.9%)	10 (4.9%)	0.645
Tricuspid valve surgery	1 (0.4 %)	0 (0.0%)	1 (0.5%)	0.706
Aortic valve minimally invasive surgery	12 (5.1%)	1 (3.4%)	11 (5.4%)	0.661
Aortic valve surgery	23 (9.8%)	2 (6.9%)	21 (10.2%)	0.571
TAVI	24 (10.3%)	3 (10.3%)	21 (10.2%)	0.987
Aortic surgery	13 (5.6%)	1 (3.4%)	12 (5.9%)	0.597
Other operations	12 (5.1%)	5 (17.2%)	7 (3.4%)	0.020
OP duration (min)	195 (157/230)	197 (147/240)	195 (158/228)	0.649
Total ventilation time (hours)	6.1 (4.4/8.6)	6.5 (5.4/11.7)	6.0 (4.3/8.5)	0.248
Bypass time (min)	83.5 (0/122.75)	83 (0/133.5)	84 (0/122)	0.875
Aortic clamping time (min)	55.0 (0/122)	40 (0/94)	56 (0/80)	0.741
Median sternotomy	164 (70.1%)	24 (82.8%)	140 (68.3%)	0.145
Minimally invasive	34 (14.5%)	2 (6.9%)	32 (15.6%)	0.213
Vascular access	24 (10.3%)	3 (10.3%)	21 (10.2%)	0.987
Partial sternotomy	12 (5.1%)	0 (0.0%)	12 (5.9%)	0.181

CABG, coronary artery bypass grafting; OP, operation; TAVI, transcatheter aortic valve implantation.

### 3.5 Implementation of the Delirium Prevention Bundle

After the implementation of the delirium prevention bundle, the use of dexmedetomidine and non-opioid analgesics, such as paracetamol or ibuprofen, increased significantly, whereas the use of zolpidem ceased completely. The administration of benzodiazepines was not significantly different after the implementation of the delirium bundle since it was already limited to patients with anxiety and agitation before this study. All medications administered before and after the implementation of the delirium bundle are shown in Table 3 (Ref. [15]).

**Table 3.** **Surgical procedures and medication behavior after implementation of the delirium bundle**.

Parameter		Number of patients before implementation (n = 116)	Number of patients after implementation (n = 118)	*p*-value
Operation				
	CABG – cardiopulmonary bypass	6	7	0.800
	Off-pump CABG	29	32	0.712
	CABG + Valve	12	13	0.868
	Valve – cardiopulmonary bypass	38	47	0.261
Medication				
	Dexmedetomidine	20 (17.2%)	51 (43.2%)	0.001
	WHO step 1 medication [15]	104 (89.7%)	114 (96.6%)	0.035
	WHO step 2 medication [15]	5 (4.3%)	3 (2.5%)	0.500
	WHO step 3 medication [15]	95 (81.9%)	105 (89.0%)	0.124
	Antidepressants	4 (3.4%)	4 (3.4%)	0.980
	Neuroleptics	4 (3.4%)	3 (3.5%)	0.684
	Benzodiazepines	5 (4.3%)	9 (7.6%)	0.285
	Zoplicone	24 (20.7%)	29 (24.6%)	0.478
	Zolpidem	5 (4.3%)	0 (0.0%)	0.023
	Metamizole	98 (84.5%)	109 (92.4%)	0.059
	Paracetamol	98 (84.5%)	111 (94.1%)	0.018
	Piritramide	90 (77.6%)	102 (86.4%)	0.078
	Oxycodone	28 (24.1%)	27 (22.9%)	0.821
	Hydromorphone	1 (0.9%)	1 (0.8%)	0.990
	Morphine	1 (0.0%)	0 (0.0%)	0.312
	Tilidine	4 (3.4%)	1 (0.8%)	0.169
	Clonidine	4 (3.4%)	3 (2.5%)	0.684
	Diclofenac	23 (19.8%)	26 (22.0%)	0.687
	Pethidine	37 (31.9%)	37 (31.4%)	0.929
	Ibuprofen	17 (14.7%)	30 (25.4%)	0.040
	Tramadol	1 (0.9%)	2 (1.7%)	0.571

WHO, World Health Organization; CABG, coronary artery bypass grafting.

Of the total analysis population studied, 11 patients (4.7%) died. The 1-year mortality was significantly higher in the delirium group with 13.8% versus 3.4% (*p* = 0.013). However, there were no statistically significant differences in in-hospital mortality (*p* = 0.706), 30-day mortality (*p* = 0.706), and 6-month mortality (*p* = 0.115). Only one patient in the non-delirium group died in the hospital within the first postoperative day.

In terms of hospital and ICU length of stay, Table 1 shows that patients with delirium stayed longer in the ICU at 1.2 days (0.8/1.2) versus 0.9 (0.8/1.0) days for patients with no delirium (*p* = 0.08) and in the hospital at 13 (12/19.5) versus 12 (11/14) days (*p* = 0.009).

We also performed stepwise bivariate logistic regression to identify independent predictors of postoperative delirium. This analysis included all the collected parameters and stepwise exclusion of non-significant parameters. The following five parameters were found to be significant: age [odds ratio (OR): 1.046; 95% confidence interval (CI): 1.002–1.092; *p* = 0.042], neuroleptics [OR: 18.758; 95% CI: 3.093–113.757; *p* < 0.001], peripheral artery disease [OR: 8.131; 95% CI: 2.336–28.306; *p* < 0.001], combined valvular defects [OR: 13.1; 95% CI: 3.240–52.974; *p* < 0.0001] and other cardiac surgery [OR: 24.149; 95% CI: 5.095–114.474; *p* < 0.001]. The parameter intervention period showed a negative regression coefficient (–0.985) in the multivariate regression calculation and the odds ratio was also less than 1 (OR: 0.374). Accordingly, significantly less delirium occurred during the period of delirium management than before implementation (Table 4).

**Table 4.** **Stepwise bivariate logistic regression model**.

Parameter	Regression coefficient B	Standard error	*p*-value	Odds ratio	95% Confidence interval for EXP (B)	
					Lower limit	Upper limit
Intervention period (delirium management with multicomponent program)	–0.985	0.501	0.049	0.374	0.140	0.998
Age	0.045	0.022	0.042	1.046	1.002	1.092
Neuroleptics	2.932	0.920	0.001	18.758	3.093	113.757
Peripheral artery disease	2.096	0.636	0.001	8.131	2.336	28.306
Combined valve defects	2.573	0.713	0.000	13.100	3.240	52.974
Other operations	3.184	0.794	0.000	24.149	5.095	114.474

EXP, exponential value.

## 4. Discussion

In our study, we showed that the implementation of delirium prevention training and the introduction of a postoperative delirium visit resulted in a statistically significant reduction in the incidence of postoperative delirium from 17.2% to 7.6%. The core idea of this study is based on that of Inouye *et al*. [10], who showed that monitoring and regular screening reduce delirium. The primary prevention of delirium is particularly effective and crucial for its treatment. For this purpose, they used a multicomponent program based on general risk factors for delirium and individual patient needs [10]. However, the intervention program showed no significant effect in decreasing the likelihood of recurrence or severity of delirium once it manifested itself [10]. Therefore, prevention is of great importance [1, 10]. This finding was confirmed in subsequent years based on the results of various studies using multicomponent programs [16]. Non-pharmacological approaches are particularly decisive in this regard, even if their influence on the duration and severity of delirium is uncertain [11]. Recommendations for further studies in this area following the successful implementation of delirium management at a general hospital by Gurlit and Möllmann [16] include education on risk factors and major clinical manifestations, early recognition of clinical signs, and implementation of screening tests as a routine measure. Luetz *et al*. [9] reported that delirium screening in ventilated patients should be considered not only as a diagnostic but also as a therapeutic measure with a treatment effect. On the one hand, they hypothesized that delirium as an early organ dysfunction may precede a systemic disease and therefore enable the early detection of sepsis. On the other hand, patient monitoring by delirium screening alone and the resulting early detection and treatment of delirium complications could have a positive impact on patient outcomes [9]. Consistent delirium screening with valid means is decisively effective in preventing delirium in both normal and intensive care units in ventilated and non-ventilated patients. Therefore, the report of a meta-analysis in which only half of all surveyed ICUs routinely screened for delirium and even <30% of all included patients were examined with a valid delirium score is all the more alarming [17]. A similarly low screening rate of 40% in the ICU was found in a survey of over 900 health care professionals in the US by Ely *et al*. [18]. In contrast, the most important component of our delirium management at the HDZ NRW was the daily bedside presence with the CAM-ICU survey as part of our post-anesthesia rounds, which ultimately significantly reduced delirium incidence at our center by ~10%. It has been known since 1985, educating nurses about the early symptoms of postoperative delirium and adequate nursing approaches for these patients have helped prevent delirium [19]. As part of the interprofessional education at HDZ, another component of our delirium management, nursing staff were introduced to regular delirium screening through post-anesthesia rounds. Memory aids distributed throughout the hospital in the form of flyers on how to perform the CAM-ICU also played a role in educating staff. After the implementation of the delirium bundle, the use of dexmedetomidine and non-opioid analgesics such as paracetamol or ibuprofen increased significantly in our study. Radtke *et al*. [20] showed that extended training leads to a higher implementation rate of therapeutic strategies for patients with delirium.

Overall, we observed a delirium rate of 12.4% in the cardiac surgery patient population in this prospective system-based quality improvement study. These results are comparable with those of previous studies with similar patient populations by authors Arenson *et al*. [21] (14.7%), Kratz [22] (11.5%) and Kazmierski *et al*. (16.3%) [23]. However, a systematic review by Gosselt *et al*. [3] reported an incidence of delirium after cardiac surgery of 2.9% to 54.9%. The incidence measured by Ely *et al*. [4] in a coronary care unit and general intensive care unit was 81.7%. In the synopsis of the results, the occurrence of delirium in our study can be classified in the lower range of reported totals A 3-day observation period was chosen because postoperative delirium manifests particularly in the first 72 hours after surgery [24]. One reason for the low delirium rate could be the avoidance of benzodiazepines preoperatively, which has been implemented at the center for quite some time according to hospital internal standards, following European recommendations for the prevention of postoperative delirium [20].

The findings of this study regarding risk factors in the multivariate analysis were age, peripheral artery disease, and combined valve defects as independent predictors of postoperative delirium. Furthermore, other rare and individual surgeries with comparatively increased surgical risks were identified as independent predictors of postoperative delirium. Bivariate bypass surgery in combination with mitral valve surgery and minimally invasive mitral valve surgery turned out to be possible triggers of delirium. Advanced age is an important known risk factor in the literature for postoperative delirium [3, 21]. In our study, patient age also showed a significant association with the occurrence of delirium in both bivariate and multivariate analyses. Bilotta *et al*. [25] provided evidence that the risk of delirium increases with age. Accordingly, patients aged >70 years have a 3-fold increased risk, and patients aged >80 have an even higher, 5-fold increased risk [25]. Several processes are observed during aging that lead to increased susceptibility to physical stress [26]. These include a decrease in neuronal plasticity and cerebral blood flow, changes in the proportion of stress-regulating neurotransmitters such as the perioperative increase in cortisol, and an increase in systemic inflammatory indicators [27]. Additionally, there is a hypothesis that cholinergic and aminergic deficits can develop in older age and thereby promote delirium [28]. Drugs with anticholinergic effects are cited as triggers, although some recent studies have refuted this association [29, 30]. In cardiac surgery, patients aged 65 and older represent approximately 60% of all cardiac surgeries [31], which is why particular caution is needed in delirium prevention in this patient population, even if age in itself represents a non-influenceable risk factor.

Combined valve surgery was identified as a risk factor for postoperative delirium in this study. The association between valve operations in cardiopulmonary and a higher incidence of delirium has been described in previous studies [32, 33]. Compared with bypass surgery, an increase in the risk of delirium has been shown [34]. In addition to postoperative delirium, this correlation has been observed in cognitive dysfunction [35]. This is caused by an increased inflammatory response due to the extent of the procedure itself and the cardiotomy performed during non-interventional valve surgery [27].

By analyzing a possible cause of another independent risk factor, summarized as the group of all other cardiac surgeries, a significantly longer hospital stay (median = 18.5 days) compared with the entire patient collective (median = 12 days) was identified. This may be because this group mainly includes high-risk procedures such as LVAD, right ventricular assist device (RVAD), or ventricular aneurysm, which may additionally explain an increased risk of delirium due to the extent of the procedure and comorbidity of patients with severe heart failure.

In multivariate analysis, peripheral artery disease was identified as the only independent risk factor among secondary diseases. A hypothesis regarding the association between generalized atherosclerosis and preoperative cognitive impairment was proposed by Vinkers *et al*. [36]. Cognitive impairment, in turn, is considered a crucial risk factor of delirium in the literature [3]. Rudolph *et al*. [37] observed that the risk of delirium increased with the degree of atherosclerosis in patients undergoing coronary artery bypass grafting. They also reported that atherosclerosis manifested itself, particularly in the frontal lobe, as features of thought disturbance and inattention belonging to the definition of delirium [37]. However, no significant differences were found for the cardiovascular risk factors of smoking, arterial hypertension, and diabetes as causes of peripheral artery disease investigated in our study.

## 5. Limitations of the Study

Although we strived to include a wide spectrum of patients undergoing cardiac surgery or interventions, the heterogeneous study population may have introduced confounding factors that were not addressed. The administration of anesthesia was not fully standardized in all cases. This lack of standardization may also have introduced confounding variables, making it challenging to isolate the effects of specific factors on patient outcomes.

## 6. Conclusions

The implementation of a delirium prevention bundle, including preoperative delirium risk stratification, multidisciplinary education, written memory aids, and post-anesthesia visits with delirium screening, demonstrated a beneficial effect on the delirium rate and was associated with improved outcomes in patients undergoing cardiac surgery or interventions. Age, double-valve surgery, and peripheral artery disease have been identified as important independent risk factors for the development of delirium. This might improve early risk stratification and strategies for this high-risk patient collective.

## Availability of Data and Materials

The dataset analyzed during the current study is available from the corresponding author on reasonable request.
